# In vivo metabolic imaging identifies lipid vulnerability in a preclinical model of Her2+/Neu breast cancer residual disease and recurrence

**DOI:** 10.1038/s41523-022-00481-3

**Published:** 2022-09-26

**Authors:** Megan C. Madonna, Joy E. Duer, Brock J. McKinney, Enakshi D. Sunassee, Brian T. Crouch, Olga Ilkayeva, Matthew D. Hirschey, James V. Alvarez, Nirmala Ramanujam

**Affiliations:** 1 Department of Biomedical Engineering, Duke University, Durham, NC USA; 2 Duke University Trinity College of Arts and Sciences, Durham, NC USA; 3 Department of Pharmacology & Cancer Biology, School of Medicine, Duke University, Durham, NC USA; 4 Duke Molecular Physiology Institute and Sarah W. Stedman Nutrition and Metabolism Center, Durham, NC USA; 5 Department of Medicine, Division of Endocrinology, Metabolism, and Nutrition, Duke University Medical Center, Durham, NC USA

**Keywords:** Cancer metabolism, Breast cancer, Cancer imaging

## Abstract

Recurrent cancer cells that evade therapy is a leading cause of death in breast cancer patients. This risk is high for women showing an overexpression of human epidermal growth factor receptor 2 (Her2). Cells that persist can rely on different substrates for energy production relative to their primary tumor counterpart. Here, we characterize metabolic reprogramming related to tumor dormancy and recurrence in a doxycycline-induced Her2+/Neu model of breast cancer with varying times to recurrence using longitudinal fluorescence microscopy. Glucose uptake (2-NBDG) and mitochondrial membrane potential (TMRE) imaging metabolically phenotype mammary tumors as they transition to regression, dormancy, and recurrence. “Fast-recurrence” tumors (time to recurrence ~55 days), transition from glycolysis to mitochondrial metabolism during regression and this persists upon recurrence. “Slow-recurrence” tumors (time to recurrence ~100 days) rely on both glycolysis and mitochondrial metabolism during recurrence. The increase in mitochondrial activity in fast-recurrence tumors is attributed to a switch from glucose to fatty acids as the primary energy source for mitochondrial metabolism. Consequently, when fast-recurrence tumors receive treatment with a fatty acid inhibitor, Etomoxir, tumors report an increase in glucose uptake and lipid synthesis during regression. Treatment with Etomoxir ultimately prolongs survival. We show that metabolic reprogramming reports on tumor recurrence characteristics, particularly at time points that are essential for actionable targets. The temporal characteristics of metabolic reprogramming will be critical in determining the use of an appropriate timing for potential therapies; namely, the notion that metabolic-targeted inhibition during regression reports long-term therapeutic benefit.

## Introduction

Breast cancer cells can survive undetected and asymptomatic for years to decades following therapy. Regrowth of these residual cells can develop into recurrent disease, the leading cause of breast cancer-related deaths^1,2^. Patients with Her2+ disease, a well-known oncogenic driver often amplified in aggressive breast cancers^3^, are more prone to relapse^4^. With the majority of all breast cancer deaths occurring between 5 and 20 years after diagnosis^2^, there is a period of tumor dormancy after initial treatment that could be a second therapeutic window to ensure that residual tumor cells are better eradicated, thus reducing the incidence of recurrence^5–8^. To identify actionable targets during this second therapeutic window, it is critical to understand how tumor cells survive during dormancy.

Studying the metabolism of these surviving cells during dormancy and upon reactivation during recurrence may identify actionable targets for treatment during the second therapeutic window, as metabolic reprogramming following environmental stress or oncogene ablation has been seen in surviving tumor cells^9–12^. It is well known that many tumors employ high rates of glycolysis even under normal oxygen conditions (i.e., the Warburg effect)^13,14^. However, some tumors can switch their primary metabolic mode between two axes of metabolism, glycolysis and oxidative phosphorylation, particularly under stressful conditions^15–17^. Further, it has become increasingly clear in the literature that the use of certain metabolites by tumor cells can change during treatment and metastasis^18^. In addition to altering the use of glucose, tumor cells can rewire their metabolism to utilize alternative fuel sources such as lactate, amino acids, and lipids^19,20^. While researchers are beginning to unravel the metabolic characteristics of cells that escape death, little is known about the underlying biology of dormant cells or residual disease surviving initial treatments^21^. In addition, the mechanisms that determine tumor dormancy duration and the requirements to activate dormant cells to return to proliferation remain poorly understood^22^.

To specifically model the treatment cascade of Her2+ tumors following targeted therapy through dormancy and recurrence, a transgenic mouse model of Her2+/Neu driven breast cancer has been widely accepted as a clinically relevant model of dormancy^23–25^. This model administers doxycycline (dox) in bitransgenic MMTV-rtTA;TetO-Her2 (MTB;TAN) mice to induce the overexpression of the oncogene Her2+/Neu, leading to the formation of mammary tumors. Her2+/Neu downregulation in this breast cancer model resembles Her2-targeted therapy often used in the clinic. Instead of Her2-targeted therapy as is used in the clinic, the removal of dox leads to Her2 downregulation and tumor regression, leaving a small population of surviving cells. This collection of cells is viable and persists in a dormant, non-proliferative state until their spontaneous recurrence.

Her2+/Neu downregulation in the aforementioned Her2+ breast cancer model has been shown to accompany an acute metabolic shift from glycolysis to oxidative metabolism^12,26–30^. Our group has previously shown that acute Her2 downregulation results in a rapid decrease in fluorodeoxyglucose ([18F]-FDG) uptake in Positron Emission Tomography (PET) in vivo in mammary tumors, immediately following treatment, irrespective of the baseline FDG uptake in the primary tumors^29^. In addition, we have shown that this reduction in glucose uptake is paired with an increase in mitochondrial metabolism in three-dimensional culture models of regressing and senescent cells^26^. Another group showed through transcriptome analysis that cell populations surviving oncogene ablation experienced a shift in metabolism towards increased fatty acid oxidation and oxidative phosphorylation compared to the primary tumor cells^30^.

Current studies are limited to in vitro or ex vivo assays or single endpoint in vivo imaging. Specifically, PET imaging does not have the resolution to specifically image the selective, highly localized alterations of a small population of dormant cells nor does it allow for multiple endpoints to be captured simultaneously^31–33^. Given that dormant cell survival and re-activation into recurrence are selective processes that are directly affected by the tumor microenvironment^1,7,34^, it is essential to understand the local variations in metabolic adaptations of residual disease in vivo by examining both axes of metabolism longitudinally. In addition, recent advances in in vitro spheroid models allow for the study of many aspects of cancer behavior like progression, treatment, associated angiogenesis, and migration^35^. The use of in vivo models allows for the direct comparisons of long-term survival during dormancy and speed of recurrence in tumors, aspects of cancer behavior not necessarily reflected in traditional in vitro models.

To address the gap in in vivo metabolic imaging, our group has previously demonstrated that glycolysis and oxidative phosphorylation can be quantified through the use of the fluorescently labeled glucose analog, 2-[N-(7-nitrobenz-2-oxa-1, 3-diazol-4-yl) amino]-2-deoxy-D-glucose (2-NBDG), to capture glucose uptake as a surrogate for glycolysis^36^, and the fluorescent cation tetramethylrhodamine ethyl ester (TMRE) to capture mitochondrial membrane potential as a surrogate for oxidative phosphorylation^37^. In addition, we have established an in vivo method for capturing both metabolic endpoints simultaneously in a window chamber model using intravital microscopy^38^. Further, our previous work reports on 2-NBDG and TMRE’s collective ability to image the dynamic changes in the metabolic landscape of regressing, residual, and re-activated breast cancer at the cellular level in vitro^26^, so it is well-suited to study small populations of dormant cells in pre-clinical models^31,32^. In vivo imaging also offers the ability to study transient changes in metabolism that will likely expand upon conclusions achieved at a single time point. Our technique is apt to study the key transitions from proliferation to dormancy and from dormancy to recurrence since repeated measurements can be taken on the same tumor as it responds to treatment or oncogene ablation. These temporal characteristics of metabolic reprogramming will be critical in evaluating the choice and appropriate timing of potential therapeutics.

This work seeks to characterize the metabolic reprogramming related to tumor dormancy and recurrence in a Her2+/Neu model of breast cancer and to identify differences in tumors that rapidly recur, modeling a highly aggressive phenotype, or tumors that persist in dormancy for a longer period of time prior to recurrence. Glucose uptake (2-NBDG) and mitochondrial membrane potential (TMRE) were imaged in mammary tumors as they transitioned to regression, through dormancy, and during recurrence. Our study was designed to provide evidence that metabolic reprogramming cannot be generalized across different phenotypes; longitudinally, the major axes of metabolism are distinctly different between tumors with varying propensities for recurrence.

“Slow-recurring” tumors (~100 days to recurrence after downregulation of Her2) increased mitochondrial metabolism during regression and dormancy and persisted at recurrence; conversely, glucose uptake decreased during regression and dormancy but then increased again upon recurrence. “Fast-recurring” tumors (~55 days) responded to Her2 downregulation with a shift from glucose to an alternative substrate source for energy in the form of fatty acid oxidation. Treatment with a carnitine palmitoyltransferase 1 (CTP1) inhibitor, Etomoxir, inhibited the switch in substrate fuel source, increased lipid synthesis, and prolonged the fast-relapsing tumors’ period of dormancy.

## Results

### Following oncogene ablation, the fast-recurring tumor showed decreased glucose uptake and increased mitochondrial membrane potential over the course of regression, dormancy, and recurrence

Her2+ breast cancers are known to modulate their metabolism during primary proliferation, regression, dormancy, and recurrence^30^. To quantify metabolic changes, female mice were orthotopically implanted with GEM (genetically engineered mice) -derived cells and given doxycycline (dox) in their drinking water to maintain Her2+/Neu expression, promoting tumor growth^23,24^. This time point is termed “Primary.” After initial tumor growth reached 5 × 5 mm, dox was withdrawn from the drinking water, leading to Her2 downregulation and a reduction in tumor volume termed “Regression.” After the regression phase, a small population of residual cells survive for an extended period of time, termed “Dormancy,” prior to re-initiating proliferation, termed “Recurrence”^39^.

We used our previously validated near-simultaneous metabolic imaging strategy of 2-NBDG (i.e., glucose uptake) and TMRE (i.e., mitochondrial membrane potential) to capture metabolic changes during these transitions. Each 2-NBDG or TMRE endpoint was captured 60 min post-injection of the respective probe and is represented by the summary parameters, 2-NBDG_60_ and TMRE_60_. Imaging time points spanned, the transition from the primary tumor to regression (8 days following dox withdrawal), followed by dormancy (28 days following dox withdrawal), and recurrence (55 days following dox withdrawal) were selected to represent each tumor phenotype.

**Fig. 1:**
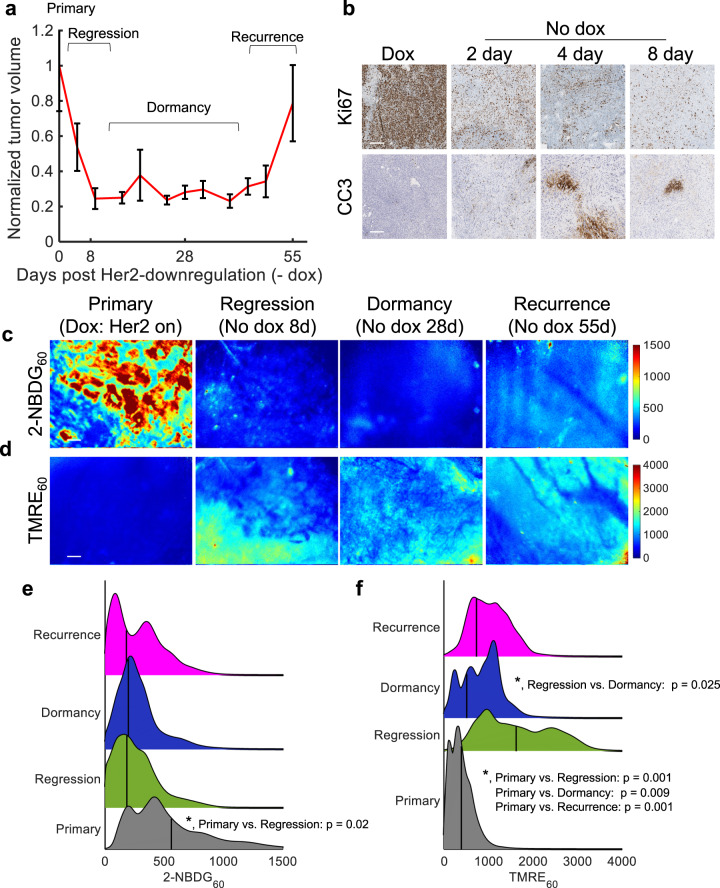
Following oncogene ablation, regressing, dormant, and recurring tumors show decreased glucose uptake and increased mitochondrial membrane potential in vivo. 2-NBDG and TMRE uptake 60 min post-injection were imaged in mammary tumors overexpressing the oncogene Her2 (+dox) and mammary tumors in which Her2 was downregulated via dox withdrawal. Recurrent tumors spontaneously re-grew ~55 days following Her2 downregulation. **a** Normalized average tumor volumes during the dormancy cycle where 0 days represents primary tumors (Her2 on). Error bar = standard error of the mean, (*n* = 11). **b** Representative immunohistochemistry images stained for Ki67 (top, proliferation) CC3 (bottom, apoptosis) (brown). **c**, **d** Representative 2-NBDG_60_ (**c**) and TMRE_60_ (**d**) images in a primary mammary tumor on dox (Her2 on) (*n* = 10), during regression (No dox 8 days) (*n* = 10), during dormancy (No dox 28 days) (*n* = 8), and upon recurrence (No dox 55 days) (*n* = 7). Scale bar = 200 µm. **e** 2-NBDG_60_ probability density function (PDF) ridgeline plots for primary, regressing, dormant, and recurrent time points across all pixels and all mice at each time point. **f** TMRE_60_ probability density function (PDF) ridgeline plots for primary, regressing, dormant, and recurrent time points across all pixels and all mice at each time point. Vertical lines superimposed on PDF curves report the average fluorescence. Statistical comparisons were performed using a Kolmogorov-Smirnov (KS) test.

Figure 1a shows the average normalized tumor volume (*n* = 11 mice) following dox withdrawal for regressing, dormant, and recurrent tumors. Tumors quickly regressed (~80% volume decrease) one-week post dox withdrawal. Tumor volume remained relatively unchanged during dormancy, followed by spontaneous regrowth 6–8 weeks after removal from dox. Along with tumor volume changes, dox withdrawal results in a significant decrease in Ki67 expression 2, 4, and 8 days after dox withdrawal (*p* < 0.05; Tukey test) and a significant increase in CC3 expression 4 days after dox withdrawal (*p* < 0.05; Tukey test) (Fig. 1b; *n* = 4 tumors, quantified in Supplementary Fig. 1a).

In vivo fluorescence imaging of window chambers following the systemic administration of 2-NBDG and TMRE allowed for the visualization and quantification of glucose uptake and mitochondrial membrane potential, respectively. Fields of view and scales for both ex vivo histology samples and in vivo fluorescence imaging were matched to better illustrate the tissue makeup and scale of the fluorescent tissue images. Further, all fields of view were entirely filled with tumor, as seen in the H&E images. Imaging of only tumor within the window ensured that the metabolic changes reported were those of the bulk tumor metabolism. Figure 1c, d shows representative images of 2-NBDG_60_ and TMRE_60_ at baseline (*n* = 10 mice) as well as during regression (*n* = 10 mice), dormancy (*n* = 8 mice), and recurrence (*n* = 7 mice). The full imaging time course is shown in Supplementary Fig. 2. The 2-NBDG signal appears higher in the Her2+ primary tumor compared to the regressing, dormant, and recurrent tumors in Fig. 1c, suggesting a decrease in glycolytic activity following Her2 downregulation. Conversely, the TMRE signal is elevated in regressing, dormant, and recurrent tumors compared to baseline, suggesting an increase in oxidative metabolism (Fig. 1d).

Probability density function (PDF) ridgeline plots were created to quantify the changes in fluorescence intensity distribution across all mice at each time point. Vertical lines superimposed on each curve report the average fluorescence for each group. Figure 1e reports a significant increase in 2-NBDG_60_ fluorescence in primary tumors compared to regressing and dormant tumors (*p* < 0.05, KS test); whereas, regressing, dormant, and recurrent tumors maintain similar levels of glucose uptake. Figure 1f shows a significant increase in mitochondrial membrane potential (TMRE_60_) in regressing, dormant, and recurrent tumors compared to primary tumors (*p* < 0.05, KS test). Further, the TMRE fluorescence progressively covers a large area within the field of view. To confirm that the dual fluorescence imaging method did not influence the metabolic changes expected in this model, the dual-injection method was compared to single probe imaging (Supplementary Fig. 3).

### Following oncogene ablation, slowly recurring tumors showed both increased glucose and mitochondrial metabolism, over the course of regression, dormancy, and recurrence

**Fig. 2:**
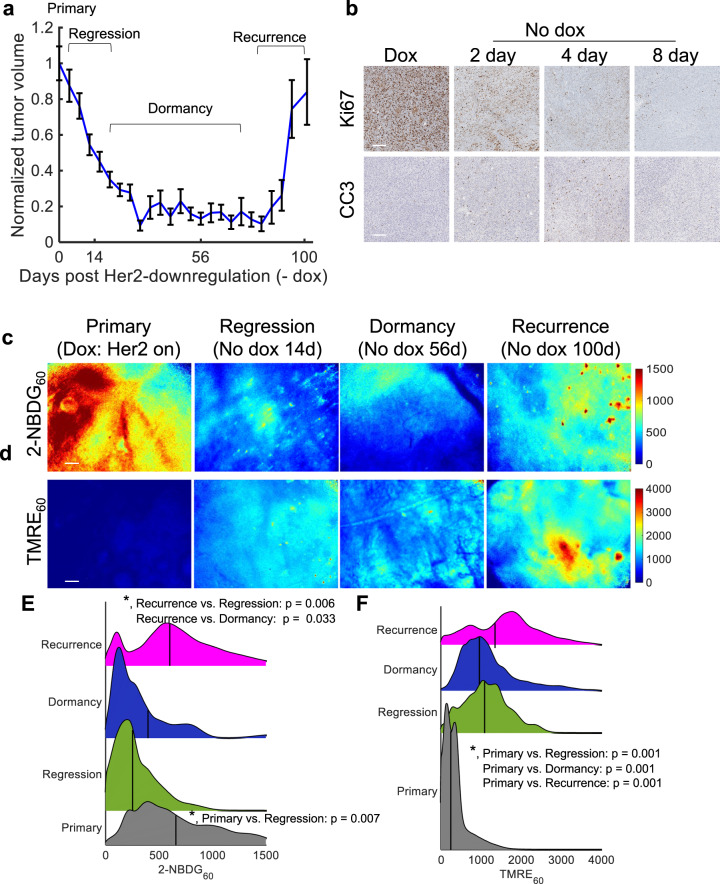
Following oncogene ablation, slowly recurring tumors showed both increased glucose and mitochondrial metabolism, over the course of regression, dormancy, and recurrence. 2-NBDG and TMRE uptake 60 min post-injection were measured in mammary tumors overexpressing the oncogene Her2 (+dox) and mammary tumors in which Her2 was downregulated via dox withdrawal. Recurrent tumors spontaneously re-grew ~90 days following Her2 downregulation. **a** Normalized average tumor volumes during the dormancy cycle where 0 days represents primary tumors (Her2 on). Error bar = standard error of the mean, (*n* = 11). **b** Representative immunohistochemistry images stained Ki67 (top, proliferation) CC3 (bottom, apoptosis) (brown). **c**, **d** Representative 2-NBDG_60_ (**c**) and TMRE_60_ (**d**) images in a primary mammary tumor on dox (Her2 on) (*n* = 10), during regression (–No dox 14 days) (*n* = 10), during dormancy (No dox 56 days) (*n* = 8), and during recurrence (No dox 100 days) (*n* = 9). **e** 2-NBDG_60_ probability density functions (PDFs) across all pixels and all mice at each time point. **f** TMRE_60_ probability density functions (PDFs) across all pixels and all mice at each time point. Vertical lines superimposed on PDF curves report the average fluorescence. Statistical comparisons were performed using a KS test.

We next investigated whether tumors that remained in dormancy longer prior to reactivation (around 90 days after Her2 downregulation, “slow-recurrence”) underwent the same metabolic shift of decreased glucose and increased mitochondrial metabolism observed in rapidly recurring tumors. Figure 2a shows the average normalized tumor volume (*n* = 11 mice) following dox withdrawal of this tumor phenotype for regressing, dormant, and recurrent tumors. Tumors regressed in approximately one month to 80% of the initial volume, with spontaneous regrowth occurring 12–13 weeks after removal of dox (4–9 weeks longer compared to the “fast-recurring” tumor line). Along with tumor volume changes, dox withdrawal in this tumor line also resulted in a significant decrease in Ki67 expression 2, 4, and 8 days after dox withdrawal (*p* < 0.05; Tukey test) and a significant increase in CC3 expression 4 and 8 days after dox withdrawal (*p* < 0.05; Tukey test) (Fig. 2b; *n* = 4 tumors, quantified in Supplementary Fig. 1b) like the fast-recurrence line.

As before, metabolic imaging was performed to capture the glucose uptake and mitochondrial membrane potential of primary, regressing, dormant, and recurrent tumors. Specifically, primary tumors (prior to dox withdrawal), regressing tumors (14 days following dox withdrawal), dormant tumors (56 days following dox withdrawal), and recurrent tumors (100 days following dox withdrawal) were selected to represent each tumor phenotype. Figure 2c, d shows representative images of 2-NBDG_60_ and TMRE_60_ at baseline (*n* = 10 mice) as well as during regression (*n* = 10 mice), dormancy (*n* = 8 mice), and recurrence (*n* = 9 mice). The full imaging time course is shown in Supplementary Fig. 4. 2-NBDG signal appears higher in the Her2+/Neu primary tumors compared to the regression and dormancy time points in Fig. 2c, suggesting a decrease in glycolytic activity even tumors are not changing in volume. Unlike the early-relapsing tumors where 2-NBDG_60_ remained decreased relative to baseline following tumor regression, the 2-NBDG_60_ signal is increased again during tumor recurrence. Conversely, the TMRE signal is elevated in regressing, dormant, and recurrent tumors compared to baseline, similar to the fast-recurrence tumors, suggesting an increase in oxidative metabolism (Fig. 2d).

PDF ridgeline plots were created to visualize the changes in fluorescence intensity distribution across each time point. Vertical lines on each plot correspond to the average fluorescence of the group. Figure 2e reports a significant increase in 2-NBDG_60_ fluorescence in primary tumors compared to regressing tumors (*p* < 0.05; KS test). In addition, after regrowth, there is once again a significant increase in 2-NBDG_60_ fluorescence compared to regressing and dormant tumors (*p* < 0.05; KS test). Similar to fast-recurrence tumors, Fig. 2f shows a significant increase in mitochondrial membrane potential (TMRE_60_) during regression, dormancy, and recurrence tumors relative to that of the primary tumors (*p* < 0.05; KS test). In addition, recurrent tumors show a further increase in TMRE_60_ fluorescence trending towards significant compared to regressing and dormant tumors (*p* < 0.06; KS test).

### Tumors varying in recurrence-free survival time show differences in glucose uptake at primary, dormancy, and recurrent endpoints

**Fig. 3:**
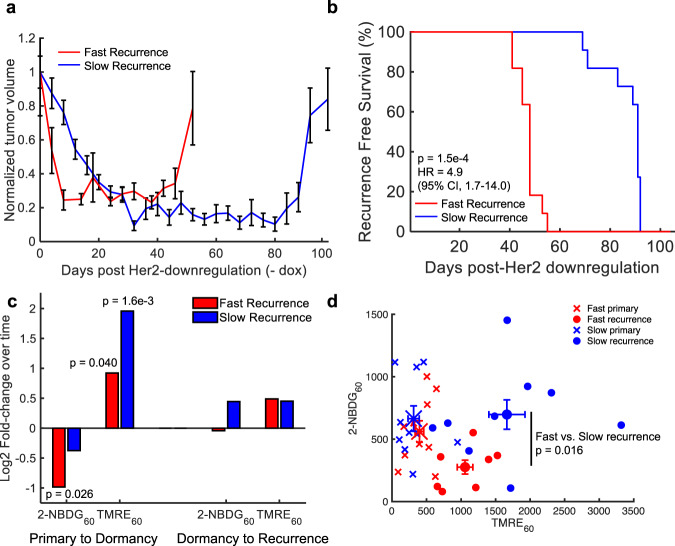
Tumors varying in recurrence-free survival time show differences in glucose uptake at the primary, dormancy, and recurrent time points. **a** Normalized average tumor volumes during the dormancy cycle where 0 days represents primary tumors (Her 2 on). Error bar = standard error of the mean. (*n* = 11 per tumor type). **b** Kaplan–Meier plot showing recurrence-free survival (RFS) for mice bearing fast or slow-recurrent tumors. Statistical comparison was determined by a Log-rank Test. (fast and slow, *n* = 11 mice). **c** Log2 fold-change in mean 2-NBDG_60_ and TMRE_60_ in primary tumors compared to dormant tumors (Log2 fold-change = Log2 (Mean_DORMANCY_/Mean_PRIMARY_)) and dormant tumors compared to recurrent tumors (Log2 fold-change = Log2 (Mean_RECURRENCE_/ Mean_DORMANCY_)). **d** Average 2-NBDG_60_ vs. TMRE_60_ for each tumor line. Statistical comparisons of fold-changes were performed with a two-sided *t*-test that compared each time point. (fast and slow primary tumor mice, *n* = 10; fast and slow dormant tumor mice, *n* = 8; slow-recurrent mice, *n* = 9 and fast-recurrent mice, *n* = 7).

We next directly compared the metabolic phenotype of the tumor lines with short and long recurrence-free survival (RFS) times to determine potential metabolic vulnerabilities that could prolong dormancy, as shown in Fig. 3a. There is a significant difference in RFS between the two tumor types (*n* = 11 per group) based on the Kaplan–Meier plot shown in Fig. 3b (*p* = 1.5e−4; Log-rank Test), corresponding to an ~83% increase in RFS in the fast-recurrence line.

We first examined whether primary tumors entering dormancy reprogrammed differently between the two tumor types. The Log2 fold-change for the transition from primary tumors (+dox, *n* = 10 mice per tumor type) to dormant tumors (fast-recurrence: off dox for 28 days, *n* = 8 mice; slow-recurrence: off dox for 56 days, *n* = 8 mice) was calculated for both 2-NBDG and TMRE and is shown in Fig. 3c. There is a significant decrease in average 2-NBDG_60_ in rapidly relapsing dormant tumors compared to that of primary tumors (*p* < 0.05; *t*-test). Conversely, there is no significant change in 2-NBDG_60_ from the primary tumor to dormancy in slowly relapsing tumors. With respect to mitochondrial membrane potential, there is a significant increase in average TMRE_60_ in both tumor phenotype groups during the transition from the primary to dormancy phase. We next investigated whether the two tumor types behaved differently as they transitioned from dormancy to recurrence by calculating the Log2 fold-change in metabolic activity between dormant and recurrent tumors (fast-recurrence: off dox for 55 days, *n* = 7 mice; slow-recurrence: off dox for 100 days, *n* = 9 mice). As seen in Fig. 3c, there is no significant change in either average 2-NBDG_60_ or TMRE_60_ when comparing both rapid and slowly relapsing tumors as they transition from their dormant to recurrent states.

Figure 3d illustrates that the primary tumors of the slow and fast-recurrent lines show similar average 2-NBDG and TMRE uptakes as well as similar levels of ATP5A1, a key subunit of ATP synthase (Supplementary Fig. 5). Both the fast- and slow-recurring tumor lines show a significant increase in TMRE_60_ uptake compared to their respective primary tumors, upon regrowth; however, the slowly-recurring cells show a significant increase in glucose uptake compared to the fast-recurrent tumors (*p* < 0.05, *t*-test), suggesting a non-glucose carbon-source is fueling the recurrence of tumors that relapse faster.

### Treatment with a fatty acid oxidation inhibitor supports a shift from glucose to fatty acids as a fuel source in the fast-recurring tumors

**Fig. 4:**
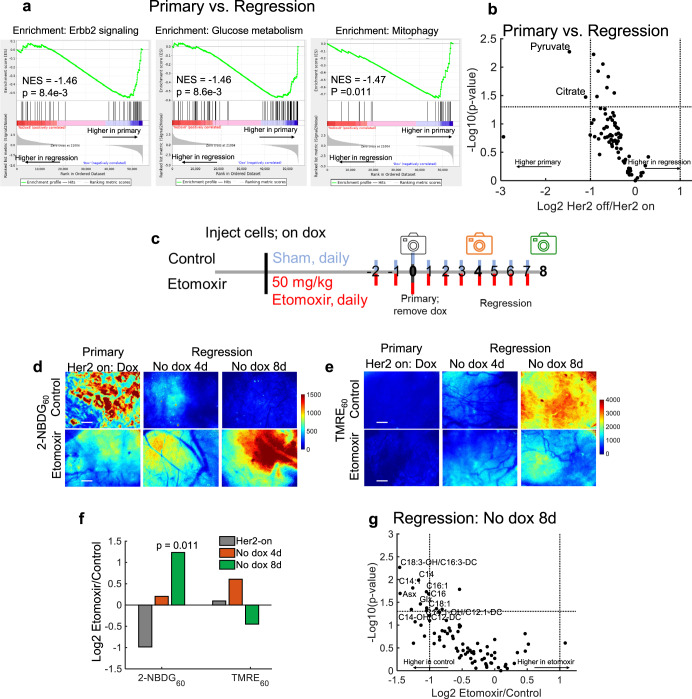
Treatment with a fatty acid oxidation inhibitor supports a shift from glucose to fatty acids as a fuel source in fast-recurring tumors. **a** Gene set enrichment analysis (GSEA) of primary (dox) and regressing (no dox 8 days) tumors. Enrichment scores were calculated using the KS statistic and *p*-values were calculated using permutation testing with 1000 permutations. **b** Volcano plot of fold-change in metabolite levels in regressing relative to primary tumors. Dashed lines represent *p* < 0.05 via two-sided *t*-test and |Log2FoldChange| > 1. Significantly different metabolites are labeled within the graph (*n* = 5 per group). **c** Schematic of treatment and imaging (camera icon) time points of control and Etomoxir treated mammary window chambers. **d** Representative 2-NBDG_60_ images of primary, Her2 on (+dox) and regressing (No dox 4 and 8 days) tumors (both untreated (Control) and treated with Etomoxir). **e** Representative TMRE_60_ images of primary, Her2 on (+dox) and regressing (No dox 4d and 8d) tumors (both untreated (Control) and treated with Etomoxir). Scale bar = 200 µm. **f** Log2 fold-change in mean 2-NBDG_60_ and TMRE_60_ in primary (Her2-on) and regressing (No dox 4d and 8d) tumors treated with Etomoxir compared with untreated tumors (control). (Log2 fold-change = Log2 (Mean_ETOMOXIR_/ Mean_CONTROL_)). Statistical comparisons of fold-changes were performed with a two-sided *t*-test between treated and control groups at each time point. (Control primary and regression tumor mice, *n* = 10; Etomoxir treated primary and regression tumor mice, *n* = 5). **g** Volcano plot of fold-change in metabolite levels between regressing tumors (no dox 8 days) with and without Etomoxir. Dashed lines represent *p* < 0.05 via two-sided *t*-test and |Log2FoldChange| > 1. Significantly different metabolites are labeled within the graph. *n* = 5 per group.

To investigate whether fatty acids were a potential carbon source towards which rapidly recurring tumors shift, we looked at genomic and metabolomic signatures. We ranked genes according to their fold-change expression between primary (+dox; *n* = 5 tumors) and regressing tumors (no dox for 8 days; *n* = 5 tumors) and performed a gene set enrichment analysis (GSEA). Figure 4a shows significant enrichment of Erbb2 (Her2) signaling (*p* < 0.01; KS test), glucose metabolism (*p* < 0.01; KS test) and mitophagy (*p* < 0.05; KS test) related gene sets in primary tumors compared to regressing tumors. In addition, primary tumors have significant enrichment of genes related to glucose metabolism compared to dormant tumors (Supplementary Fig. 6a). Figure 4b shows metabolomics results at the same time points (*n* = 5 tumors per time point), which show that pyruvate and citrate are significantly decreased in regressing tumors compared to primary tumors (*p* < 0.05 and |Log2FoldChange| > 1). Similarly, dormant tumors had significantly decreased pyruvate levels compared to primary tumors (Supplementary Fig. 6b).

The genomic, metabolomic, and imaging results indicate a shift from glycolysis in regressing tumors in which Her2 has been downregulated. Further, imaging results indicate a decrease in glycolysis, and an increase in mitochondrial respiration in recurrent tumors. We investigated whether fatty acids served as an alternative carbon source in rapidly recurring tumors during the period of tumor regression. We treated primary and early regressing tumors daily for 10 days with a known fatty acid oxidation inhibitor, Etomoxir^40^, or sterile saline (sham), beginning 2 days before baseline imaging (i.e., before dox withdrawal) and continuing with concomitant imaging until 8 days after dox withdrawal, as depicted in Fig. 4c. Figure 4d,e shows representative 2-NBDG_60_ and TMRE_60_ images, respectively, of control (untreated, *n* = 10 mice) and Etomoxir-treated mice (*n* = 5 mice) at baseline (Her2 on) as well as 4- and 8-days post dox withdrawal. Mice treated with Etomoxir have higher 2-NBDG uptake through tumor regression than untreated controls. While TMRE_60_ increases significantly during tumor regression in the control group, this is less pronounced in the treated group. Figure 4f shows the Log2 fold-change in mean 2-NBDG_60_ and TMRE_60_ of tumors treated with Etomoxir relative to control tumors. There is a significant increase in 2-NBDG_60_ 8 days following dox withdrawal in Etomoxir-treated tumors relative to control tumors (P < 0.05; *t*-test). Though there is no significant change in average TMRE_60_ when comparing Etomoxir-treated tumors with control tumors at each time point, TMRE_60_ does show a decreasing trend in the treated group 8-days post dox withdrawal. To ensure the Etomoxir treatment inhibited long-chain fatty acids from entering the mitochondria, thus preventing oxidation, the metabolomic analysis compared metabolite levels of the treated and control tumors at 8-days post dox withdrawal. Figure 4g shows that Etomoxir treatment led to significantly decreased levels (|Log2FoldChange| > 1, *p* < 0.05; *t*-test) of 8 acylcarnitines and 2 amino acids compared to that of regressing tumors in the control group. Taken together, the imaging and metabolomics results indicate Etomoxir targets lipid metabolism in the regressing tumor.

### Inhibiting fatty acid oxidation in regressing tumors impairs recurrence and increases lipid synthesis

**Fig. 5:**
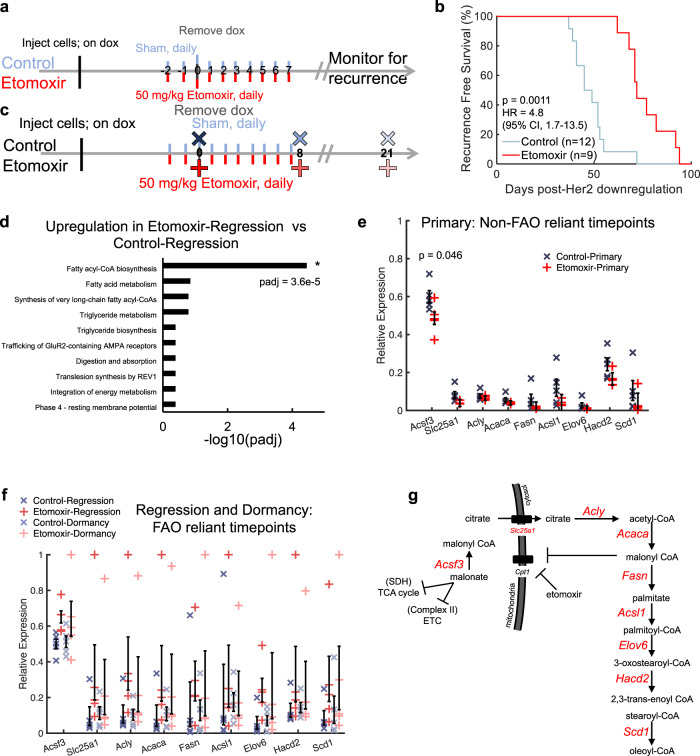
Inhibiting fatty acid oxidation in regressing tumors impairs recurrence and increases lipid synthesis. **a** Schematic of recurrence assay comparing control and Etomoxir-treated mice. **b** Kaplan–Meier plot showing recurrence-free survival (RFS) between untreated tumors (Control) and tumors treated with Etomoxir. Statistical comparison was determined by a Log-rank Test. (Control mice, *n* = 12; Etomoxir-treated mice, *n* = 9). **c** Schematic showing time points of tumor harvest for RNA sequencing. **d** Gene set over-representation enrichment analysis comparing Etomoxir and control regressing tumors. Statistical comparisons of enriched pathways were performed using the Wald test followed by the Benjamini and Hochberg’s correction for multiple comparisons. **e** RNA sequencing analysis of fatty acyl-CoA biosynthesis-related gene set in primary tumors (non-fatty acid oxidation (non-FAO)-reliant time points). (*n* = 5 tumors per group). **f** RNA sequencing analysis of fatty acyl-CoA biosynthesis-related gene set in regressing and dormant tumors (FAO-reliant time points). (*n* = 5 tumors per group). Error bar = standard error of the mean. Statistical comparisons of expression levels were performed with an ANOVA followed by a Tukey test for multiple comparisons. **g** Schematic showing transcriptional regulation of fatty acid synthesis and elongation where genes quantified in (**e**) and (**f**) are shown in red. SDH succinate dehydrogenase, ETC electron transport chain.

After verifying that Etomoxir treatment decreased the fatty acid oxidation signature, we investigated whether this metabolic inhibition affected survival. A parallel cohort of mice received the same treatment (10 days) as those imaged acutely and was followed for survival (Fig. 5a). Tumors were palpated 3 times per week to monitor for the emergence of recurrent tumors. Etomoxir treated mice (*n* = 9 mice) recurred more than 3 weeks later than control mice (*n* = 12 mice) (Fig. 5b; *p* = 0.0011, Log-rank test). This corresponded to a ~59% increase in time for RFS. Treating mice with a fatty acid oxidation inhibitor prolonged the periods of long-term dormancy and recurrence-free survival in these mice.

As a first step towards understanding how fatty acid oxidation inhibition extended tumor-free survival, RNA sequencing of primary, regressing, and dormant (mid-way through expected recurrence in control) tumors treated with (red) and without (blue) Etomoxir was performed (Fig. 5c). When specifically comparing the over-representation of gene sets between the Etomoxir and control regression time points, the only significantly enriched gene set in the Etomoxir-treated tumors (*n* = 5 per group) was genes related to fatty acyl-CoA biosynthesis (*p*_adj_ < 0.05; Wald test with Benjamini and Hochberg correction) (Fig. 5d). This same regression time point is where 2-NBDG_60_ was significantly higher following treatment. To examine if this observation was specific to tumors reliant on fatty acid oxidation (oncogene ablated tumors), mRNA expression levels of each gene (7 total) within this gene set plus two additional key genes within the fatty acid synthesis pathway (*Fasn, Acsl1*) was compared. The primary time points (reported to rely on glucose metabolism) show little differences in gene expression between the treated and control tumors (Fig. 5e; *n* = 5 tumors per group). This is in contrast to regressing and dormant tumors. Regressing and dormant time points, reported in Fig. 5f, are perceived to rely on fatty acid oxidation. The mRNA expression levels of both regressing and dormant tumors treated with Etomoxir show an increase in key fatty acid biosynthesis genes compared to the control tumors (Fig. 5f) (*n* = 5 tumors per group). The difference in response to Etomoxir in the primary tumors compared to the regressing and dormant tumors further point to the treatment’s specificity for fatty acid-fueled tumors. Figure 5g depicts the fatty acid synthesis and elongation-related genes plotted in Fig. 5f, indicating the pathway-wide effect Etomoxir plays in regressing and dormant tumors.

## Discussion

An undetected residual disease that escapes primary treatment serves as a reservoir of cancer cells with the propensity to recur^41,42^. It is crucial, therefore, to study these cells specifically to understand and prevent future recurrence. Towards this goal, we have used in vivo imaging methods to simultaneously image 2-NBDG and TMRE^38^ in a well-known Her2+/Neu GEM model of dormancy and recurrence to pinpoint metabolic signatures of breast cancer primary, regressing, dormant, and recurrent tumors. Through these experiments, our platform was able to characterize metabolic reprogramming that accompanies Her2 downregulation as tumors shrink, persist as residual disease, and ultimately spontaneously recur as would occur in patients. Collectively, glucose uptake (2-NBDG) and mitochondrial membrane potential (TMRE) identified differences in metabolism between tumors that rapidly recur, modeling a highly aggressive phenotype, or tumors that persist in dormancy longer before recurrence, independent of mitochondrial mass alone. Beyond this, imaging was able to pinpoint the timing where genomic and metabolomic approaches could delve into the underlying behavior of these cells.

There are several techniques across various length scales that have been designed to report on metabolic reprogramming^33,43–46^, but there is a gap in technologies that allow for the direct longitudinal imaging of two key axes of metabolism (glycolysis and oxidative phosphorylation) at the same time in orthotopic preclinical models. Advances in PET technology have allowed for the imaging of both glucose uptake and mitochondrial membrane potential in the same animal over the course of 24 h^33^; however, this work and previous work highlight the time-dependence of metabolic reprogramming throughout a tumor’s dormancy cycle, suggesting a need for simultaneous imaging of endpoints longitudinally^26,47^. In addition, with a spatial resolution of 1–2 mm, PET imaging may miss metabolic changes in small populations of dormant cells^31,32^. Our optical imaging methodology aims to fill this gap and is poised to address the question of metabolic reprogramming in residual tumor cells that persist in dormancy. Given previous reports of metabolic changes in breast tumors following Her2 downregulation, we proposed to capture the balance between glycolysis and mitochondrial metabolism and demonstrate how the balance of both could shift throughout a tumor’s dormancy cycle^12,26–29^.

This work demonstrates that metabolism is an important marker of study for comparing tumor aggressiveness. We observed transient metabolic shifts throughout the tumor’s regression, dormancy, and recurrence. Specifically, we found that primary Her2+/Neu tumors heavily relied on glucose fermentation (“Warburg Effect”)^26,29^. This metabolic phenotype shifts following Her2 downregulation indicate a higher reliance on the mitochondria for energy through oxidative phosphorylation for the cells surviving oncogene ablation. This is consistent with results reported by others in similar oncogene addiction models following ablation^12,30^.

This difference in metabolic adaptation following Her2 downregulation between recurrent tumors that vary in latency alludes to an alternative source uniquely fueling the aggressive phenotype’s reactivation for recurrence not seen in the slow-recurrent tumor type. In addition, this change in mitochondrial activity levels does not appear to be a function of mitochondrial mass within each tumor type alone. Through in vivo imaging, we highlight a clinically actionable target for these rapidly recurring cells. Given this unique phenotype seen only in the fast-recurrence cells, we hypothesized that fast-relapsing tumors increased their use of fatty acids following Her2 downregulation as previous work has reported in a myriad of cancer types^12,30,47^. Further, previous work has provided evidence to support that adipose tissue surrounding tumors may drive a tumor’s aggressiveness by providing exogenous fatty acids to the cancer cells^48^.

We sought to defend this hypothesis of the lipid dependence through additional assays. Specifically, fatty acid oxidation of the fast-recurrence tumor line was targeted with Etomoxir, as it is considered one of most widely used fatty acid oxidation inhibitors^40^. Following treatment with Etomoxir, surviving tumor cells were reprogrammed to again rely on glucose (increased 2-NBDG_60_) similar to the primary tumors; however, primary tumors show no change in 2-NBDG_60_ or TMRE_60_ following Etomoxir treatment. This increase in 2-NBDG_60_ in regressing cells is in agreement with previous studies that report the ability of Etomoxir to increase glucose uptake in highly lipid-dependent normal and tumor tissues^49,50^. When examining mitochondrial activity, however, TMRE_60_ was not significantly different during regression in Etomoxir-treated compared to control tumors. Further, a parallel cohort of Etomoxir-treated and control tumors was analyzed with metabolomics. Regressing cells treated with Etomoxir contained significantly decreased levels of acylcarnitines, indicating increased lipid catabolism in the control cells. It is important to note, however, that the use of Etomoxir and other fatty acid oxidation inhibitors has shown off-target effects by disrupting mitochondrial pathways more broadly, which could have also contributed as a potential therapeutic target for the cells and thus, should be noted as a limitation of this study. Because of this, in the future it is vital to continue metabolic studies to better pinpoint more specific metabolic pathways as potential druggable targets that limit off-target side effects as well as the creation of highly specific therapeutic options.

This work also highlights the importance of the timing of metabolic changes through regression, deepening the necessity of longitudinal imaging to capture these changes. Here, we saw that the resurgence of glucose uptake to levels matching primary tumors in etomoxir-treated regressing tumors was not observed until 10 days of treatment (2 days before Her2 downregulation through 8 days following inhibition). Before this time point, at 4 days following Her2 downregulation, the increase in glucose uptake was not observed, underscoring the importance of capturing transient changes as well as the time points at which tissues can be harvested for additional assays.

The fatty acid oxidation inhibition not only alludes to a change in metabolite (fatty acids back to glucose) use but also highlights the continued need for mitochondrial metabolism (sustained TMRE_60_ signal) to facilitate fuel production during the period of tumor regression. Although these results support the growing hypothesis of the importance of fatty acid oxidation as a fuel source for many tumors, many studies also indicate the importance of mitochondrial metabolism more generally^9,51^. Even in studies such as those described here, in lieu of inhibiting fatty acid oxidation through Etomoxir, inhibiting oxidative phosphorylation or the electron transport chain could also lead to a similar result while also preventing the use of other nutrients such as pyruvate, stromal-derived lactate, and glutamine, likely leading to a significant decrease in TMRE_60_ as well.

As TMRE serves as a metric for mitochondria membrane potential, multiple variables, pathways, and environments may affect TMRE compared to 2-NBDG, which directly measures glucose uptake. Using both together allow for a larger picture of the two main axes of metabolism^37^. Because TMRE does not report on specific carbon inputs fueling the electron transport chain, measuring additional specific inputs would be required to directly inform which alternative fuel sources are vital for dormancy and recurrence through our techniques. To overcome this limitation, further studies including additional fluorescence markers for fatty acid and amino acid uptake could be included to robustly report on specific metabolic reprogramming^52,53^.

Our longitudinal imaging technique indicates that mitochondrial metabolism plays a major role in regressing disease following Her2 downregulation and suggest an actionable target for future therapy. Although similar metabolic changes have been demonstrated in primary tumor cells and residual disease through in vitro cultures of tumor cells in isolation^26^, many groups have also reported on the importance of the tumor microenvironment and its metabolism in recurrence^54–56^. Because of this finding and because this study reports only on the bulk metabolism of the entire field of view, future high-resolution imaging using cell-specific markers should be explored to report on the metabolic interplay of the tumor and stromal cells to delineate each cell type’s role.

Interestingly, aside from acute metabolic changes, blocking fatty acid transport into the mitochondria while tumors shrank into dormancy prolonged the period of dormancy by nearly 60%. This supports the case for fatty acid oxidation as a potential therapeutic target for breast cancers with a high risk of rapid recurrence. This prolonged dormancy could be due to (1) the exploitation of the tumor’s metabolic vulnerability, thus reducing the number of tumor cells surviving into residual disease thereby creating a larger period of dormancy before evidence of regrowth, (2) the reprogramming of these regressing tumors to a less aggressive phenotype similar to the slow-recurrent tumors that have a longer dormancy period (increased use of glucose), or (3) a combination of the two hypotheses.

Nevertheless, detailing the mechanism by which this treatment prolongs dormancy may hold clues to its application to a wide range of tumors, so we probed the transcription and metabolomic changes following the addition of Etomoxir. RNA sequencing indicated the only significant over-represented gene set in the Etomoxir-treated regressing cells compared to the regressing tumors in the control group were genes involved in fatty acid synthesis and elongation. In addition, this pattern is not observed in the primary tumor groups where fatty acid oxidation is inferred to not be a primary source of fuel. This increase in fatty acid synthesis may be due to the cells’ need to maintain their energy balance and reduce lipid toxicity from increased accumulation^57–59^. Further, within the fatty acid synthesis pathway, the first, irreversible step is the carboxylation of acetyl-CoA to malonyl-CoA, an inhibitor of Cpt1^60^. This may specifically compound the effects of the Etomoxir treatment, though future work is required to substantiate this potential additional mechanism of action. Given the observation of prolonged dormancy in tumor cells undergoing increased fatty acid synthesis and elongation, our work supports growing evidence of the importance of lipid metabolism in the context of dormancy and recurrence^61,62^.

While cytoplasm-bound malonyl-CoA has been well documented and studied through its role in lipid metabolism, the malonyl-CoA generated within the mitochondrial matrix is less explored. Our transcriptional analysis indicated Etomoxir treatment additionally causes increases in *Acsf3* expression, the gene responsible for producing malonyl-CoA from malonate within the mitochondria, in regressing and dormant tumors. Because malonate is a known succinate dehydrogenase and Complex II inhibitor, this increase in *Acsf3* expression may further confirm our imaging results indicating the continued use of mitochondrial respiration as fuel even following Etomoxir treatment^63,64^. Finally, this transcriptional signature of increased fatty acid synthesis-related genes extends beyond time points directly treated with Etomoxir (i.e., dormant tumors), which may explain the chronic phenotypic changes of the extended survival following an acute treatment regime.

Although there has been an improvement in cancer diagnosis and treatment, the recurrence of cancer cells that previously evaded therapy remains a leading cause of death in patients with cancer^42^. Cells that persist as residual disease and spontaneously recur can be metabolically unique from their respective primary tumors^65,66^. In summary, our technology and model links spatial and temporal signatures of metabolism (glucose uptake and mitochondrial membrane potential) to key time points during a tumor’s dormancy cycle providing an opportunity to evaluate residual cell treatment to prevent tumor relapses. Due to limitations of existing in vitro, ex vivo, or in vivo metabolic assays, these observations can be laborious and require large animal cohorts. The use of this technique allowed for observation of in vivo repeated, real-time metabolic phenotyping across a tumor’s dormancy cycle. This allowed for key time points to be pinpointed for further observations and exploited to prolong tumor dormancy in tumors prone to recurrence.

Breast tumors are an excellent system for studying tumor dormancy since dormancy is well described clinically in breast cancer. Given the high incidence of breast cancer and its prolonged recurrence time, a large number of women are living with dormant, residual disease that is at risk of recurring^2,5,67^. The genetically engineered model of oncogene ablation chosen for this work is the only model we know of in which residual cells persist in a dormant, non-proliferative state for an extended period^42^. It should be noted that this model is limited to modeling local recurrence and that distant metastases presented in the clinic may differ from the phenotype of local recurrences; however, because local recurrences have been shown to be strongly associated with the risk and timing of distant recurrence, we elected to image and report on the metabolic characteristics of the locally recurrent tumors^42,68–70^. Because of this, additional examination of this potential lipid dependency seen in local recurrent tumors should be confirmed in metastatic recurrent models for potential clinical translation. To avoid this limitation, future work in the field of dormancy imaging should include the validation and implementation of dormancy and recurrence models using window chambers of distant locations, such as lung, liver, and brain windows to allow for direct metabolic imaging of distant tumor sites and to better model clinical recurrence^71,72^.

Further, several factors including genetic, epigenetic, microenvironmental pathways, and metabolic pathways can all play a role in tumor dormancy and recurrence and not all of these observed in an induced Her2+/Neu model can faithfully model those seen in clinical recurrence^54–56,61,73,74^. Finally, it is important to note a clear distinction between the Her2+/Neu oncogene ablation model and clinical Her2+ tumors. Patients being treated for Her2 breast cancer typically receive anti-Her2 and chemotherapies, whereas the oncogene ablation model used in this work models this through the downregulation of the Her2+/Neu oncogene. This cannot completely recapitulate all mechanisms that tumor cells alter to survive.

In this work, we highlight the importance of longitudinally monitoring glucose uptake and mitochondrial membrane potential during dormant cell survival and recurrence and report how altered cellular metabolism is a downstream consequence of diverse upstream pathways. Because of this, studying tumor cell metabolism can inform on dormancy induced by oncogene ablation. Therefore, this work offers the ability to capture metabolic vulnerabilities in vivo through intra-vital, multi-parametric imaging to inform on a tumor’s metabolic landscape.

## Methods

### Animal studies

All in vivo murine experiments received ethical approval by Duke University’s Institutional Animal Care and Use Committee (IACUC), were conducted according to Protocol A072-18-03, and are compliant with all relevant ethical regulations for animal testing and research. All mice were housed 5 mice per cage in an on-site housing facility with ad libitum access to standard laboratory food and water with a standard 12/12 light cycle. Heat and humidity were maintained within the parameters outlined in The Guide for the Care and Use of Laboratory Animals. Mice were housed in micro-isolator caging with corn cob bedding. Cages were changed once per week.

### Genetically engineered Her2-overexpressing murine model

To create the genetically engineered derived tumor models, doxycycline-dependent Her2+/Neu breast tumors were harvested from MMTV-rtTA; TetO-NeuNT (MTB;TAN) mice. Following, tumors were minced and digested at 37 °C in Earle’s Balanced Salt Solution (EBSS) without phenol red media (Gibco 14155-063) supplemented with 300 U/mL collagenase and 100 U/mL hyaluronidase (StemCell 7912), 2% FBS (MediaTech 35-010-CV), 100 µg/mL gentamycin (Sigma G1264), 1% PenStrep (Gibco 15140-122) and 2 µg/mL doxycycline (RPI D43020) to maintain expression of Her2. Cell lines were regularly monitored for contamination and tested for mycoplasma.

For propagation, dissociated tumor cells were cultured using DMEM media (GenClone 25-501) supplemented with 10% Super Calf Serum (Heat inactivated; Gemini 100-510), 1% PenStrep (Gibco 15140-122), 1% L-Glutamine (Gibco 25030-081), 10 ng/ml murine EGF (Sigma E4127), 5 ug/ml bovine insulin (Gemini 700-112 P), 1 µM progesterone (Sigma P7556), 1 µg/ml hydrocortisone (Sigma H0396), and 5 µg/ml ovine prolactin (NIDDK-oPRL-21), and 2 µg/mL doxycycline (Dox) (RPI D43020) to maintain expression of Her2. To passage, cells were collected, spun down, and trypsinized for 5 min. Trypsin was quenched using whole media, and cells were split 1:3. Cells were passaged every 3 days.

Two previously established primary tumor cell lines were used in this study^75^. The in vivo primary and recurrent tumor behavior of both primary tumor cell lines has been previously established as one primary cell line prone to recur ~55 days following doxycycline withdrawal (referred to as: ‘fast-recurrence’) and one primary cell line prone to recur ~100 days following doxycycline withdrawal (referred to as: ‘slow-recurrence’).

For orthotopic injection, the abdomen of 6–8-week-old female nu/nu (Charles River) mice was cleaned using 70% ethanol and the 4th mammary gland was palpated and injected with 100 μL (~5 × 10^5^ cells) of ‘fast-recurrence’ cells prone to recur ~55 days following doxycycline withdrawal or 100 μL (~1 × 10^6^ cells) of ‘slow-recurrence’ cells prone to recur ~100 days following doxycycline withdrawal using a 27 G needle.

Mice were supplied with 2 mg/mL dox water to induce Her2+ tumor growth. Tumor volumes were measured 3 times per week using calipers and calculated as Volume = (Length × Width^2^)/2, where width represents the smallest axis and length the longest axis. Once tumors reached 5 mm in width, dox was removed to induce Her2 downregulation and tumor regression. For etomoxir studies, primary and early regressing tumors were treated daily for 10 days with a known fatty acid oxidation inhibitor, Etomoxir^40^, or sterile saline (sham), beginning 2 days before dox withdrawal until 8 days after dox withdrawal. For recurrence studies, dormant tumors were considered recurrent following two consecutive increasing measurements in relation to prolonged dormancy measurements. Tumor monitoring and volume measurements were collected by a blinded researcher.

### Murine mammary window chamber

Prior to surgery, mice were anesthetized using isoflurane (1–1.5% v/v) in room air in an induction chamber. The animal was transferred to a heated, sterile surgical field to maintain core body temperature and sterility during surgery. Mice were administered 1 ml/kg Buprenorphine SR via subcutaneous injection for pain relief. Titanium window chambers with 12 mm diameter, No. 2 glass coverslips were surgically implanted over the 4th right mammary gland of female nu/nu (Charles River) mice using an established procedure^76^ at key points of the tumor life cycle with all tumors remaining under 150 mm^3^. Upon tumors achieving a tumor size of 5 × 5 mm, tumors were randomly assigned imaging groups using a random number generator. Tumors modeled either a primary tumor (doxycycline present, actively growing in volume; *n* = 10 rapid relapse tumor mice, *n* = 10 slow relapse tumor mice), a regressing tumor (doxycycline removed <15 days prior to imaging, actively shrinking in volume; *n* = 10 rapid relapse tumor mice, *n* = 10 slow relapse tumor mice), a dormant tumor (doxycycline removed 21 or 56 days prior to imaging, constant volume; *n* = 8 rapid relapse tumor mice, *n* = 8 slow relapse tumor mice), recurrent tumor (doxycycline removed 55 or 100 days prior to imaging, actively growing in volume; *n* = 7 rapid relapse tumor mice, *n* = 9 slow relapse tumor mice). For replication purposes, each imaging time point was collected on, at least, two different days. Mice were allowed >24 h to recover from surgery prior to imaging experiments. Primary and regression tumor timepoints were captured using the same cohort of mice. Dormancy and recurrent timepoints were captured using unique cohorts given the viability of window chambers (~2 weeks following surgery). Following a mouse’s final imaging timepoint, mice were euthanized by isoflurane inhalation overdose. Euthanasia was confirmed using a bilateral thoracotomy.

### Imaging probes

For in vivo administration, TMRE (Tetramethylrhodamine Ethyl Ester, Life Technologies/ThermoFisher) was diluted to a final concentration of 75 μM in sterile phosphate-buffered saline (PBS), and 2-NBDG (2-(*N*-(7-Nitrobenz-2-oxa-1,3-diazol-4-yl)Amino)-2-Deoxyglucose, Duke University Small Molecule Facility) was diluted to a final concentration of 6 mM according to previously established protocols^38^. For simultaneous imaging with both probes, 100 μL of 75 μM TMRE was injected first, and then 100 μL of 6 mM 2-NBDG was injected second, 15 min later. Both probes were delivered systemically via retro-orbital injection.

### Fluorescence microscopy system and metabolic imaging

All mice were fasted for 4 h (water provided) prior to imaging to ensure a normalized metabolic rate for each animal^77^. Following fasting, imaging began immediately. The animal was anesthetized using isoflurane (1–1.5% v/v) in room air in an induction chamber. The animal was transferred to a heated microscope stage (to maintain core body temperature) where a background image (laser on) and dark image (laser off) were acquired prior to probe administration to account for the signal from sources other than the fluorescence probes. Both TMRE and 2-NBDG imaging was acquired 60 minutes following their respective injection.

An exposure time of 5 s was used for TMRE images, and an exposure time of 2 s was used for 2-NBDG images. To account for daily light source variation, all images were background subtracted, beam shape corrected, and calibrated according to a Rhodamine B standard imaged at each imaging session prior to image analysis.

A custom-built, previously reported microscope^38^ was used to capture fluorescence for both metabolic endpoints. For TMRE imaging, a 555 nm crystal laser (CL555-100-O, Crystal laser, Reno, NV, USA) was used to excite the probe, followed by a 573 nm longpass dichroic mirror (FF573-Di01-25 × 36, Semrock, Rochester, New York, USA) to reject the excitation light in the emission channel. Emission signal was collected using a liquid crystal tunable filter (LCTF) (VariSpec VIS-7-35, PerkinElmer, Inc. Waltham, MA, USA, 7 nm bandwidth) programmed to collect at 585 nm and a high-resolution dual-modal charge-coupled device (CCD) (ORCA-Flash4.0, Hamamatsu, Japan).

For 2-NBDG imaging, a 488 nm crystal laser (DL488–100-O, Crystal laser, Reno, NV, USA) was used to excite the probe, followed by a 505 nm longpass dichroic mirror (DMLP505R, Thorlabs, USA) to reject the excitation light in the emission channel. The emission signal was collected using the previously described LCTF (programmed to collect at 545 nm) and CCD. The spectral microscope system was calibrated wavelength by wavelength using a standard lamp source (OL 220 M, S/N: M-1048, Optronic Laboratories, USA).

This system uses a Nikon CFI E Plan Achromat 4x objective (NA = 0.1, Nikon Instruments Inc., USA) for all imaging. This creates a single frame field of view of 2.1 mm × 1.6 mm and a lateral resolution of 2.2 µm, as measured using a 1951 USAF resolution target^38^. This microscope was controlled by a custom-designed LabVIEW software.

### Optical imaging data processing and statistical analysis

All post-processing and statistical analysis were performed using MATLAB (MathWorks, USA). Prior to further analysis, each image collected first underwent background and dark noise subtraction by removing the average value of an image collected with the laser source off (dark noise) and removing the average signal imaged prior to fluorescent probe injection (background). In addition, due to the non-uniformity of illumination of a Gaussian-shaped light source, each image was divided by an image of a uniform phantom to correct for the beam shape. Finally, each image was calibrated using a Rhodamine B standard solution imaged during each imaging session. These methods accounted for autofluorescence, non-uniform illumination, and day-to-day system variations, respectively. The resulting images were used for all statistical comparisons and images displayed in this study. Sample sizes were selected based on previously published work to attain 80% power at a 5% level of significance^37^. Analysis was performed by non-blinded individuals.

Probability density functions (PDFs) were chosen to visualize the changes in fluorescence intensity distribution across entire images between different metabolic phenotypes using the summary parameter, 2-NBDG_60_ or TMRE_60_, representing the fluorescence that is present 60 min post-injection. Empirical *p*-values were calculated for a Kolmogorov-Smirnov statistic using blocked permutation (*n* = 1000 random permutations per test) to compare distributions, prior to binning data for graphing^78^. In addition, a comparison of average intensities of 2-NBDG_60_ or TMRE_60_ was performed using a one-way ANOVA followed by a Tukey test for multiple comparisons. Log fold-changes between time points were calculated as Log2 fold-change = Log2 (Mean_TIMEPOINT1_/Mean_TIMEPOINT2_). *P*-values < 0.05 were considered significant and exact *p*-values are reported within each figure.

### Immunohistochemical staining

Mammary tumors were grown to a size of 5 × 5 mm. Tumors were randomly assigned time points using a random number generator. Prior to excision, mice were anesthetized using isoflurane (1–1.5% v/v) in room air in an induction chamber. The animal was transferred to a heated, sterile surgical field to maintain core body temperature and sterility. At each time point, mammary tumors were harvested from mice, fixed with 4% paraformaldehyde in PBS, and stained for Ki67 (RM-9106-S, ThermoFisher, 1:200), CC3 (D3E9 #9579, Cell Signaling, 1:200), or ATP5A1 (PA5-25704, ThermoFisher, 1:1500). Following excision, mice were immediately euthanized by isoflurane inhalation overdose. Euthanasia was confirmed using a bilateral thoracotomy. A sample size of four tumors per group was used. Expression was quantified using Fiji. Statical comparisons were performed using a one-way ANOVA followed by a Tukey’s test for multiple comparisons. Analysis was performed by non-blinded individuals.

### RNA sequencing

Mammary tumors were grown to a size of 5 × 5 mm. Tumors were randomly assigned time points using a random number generator. Prior to excision, mice were anesthetized using isoflurane (1–1.5% v/v) in room air in an induction chamber. The animal was transferred to a heated, sterile surgical field to maintain core body temperature and sterility. At each time point, mammary tumors were harvested from mice and flash-frozen. Following excision, mice were immediately euthanized by isoflurane inhalation overdose. Euthanasia was confirmed using a bilateral thoracotomy.

All RNA sequencing was completed by Novogene (Durham, NC). RNA sequencing tumor samples and datasets were prepared and created by a blinded individual. Briefly, directional library preparation with rRNA depletion was performed and libraries were sequenced on NovaSeq 6000 with PE150 reads. A sample size of five tumors per group was used. Quantified expression levels were plotted as bar graphs by normalizing each level according to the maximum expression value for a given gene. Expression levels were then averaged across each group. For pathway analysis, the Reactome pathway database determined significantly enriched pathways using DESeq2 (R package 1.20.0) and *p*-values were determined using a Wald test followed by the Benjamini and Hochberg’s approach to account for multiple comparisons and control for a false discovery rate. Genes with an adjusted *p*-value of <0.05 were considered significant and exact *p*-values are reported within each figure. Further, Gene set enrichment analysis (GSEA) was calculated to determine enriched pathways between tumor phenotypes. Differences in gene expression were calculated between a given two-group comparison and ranked. “Signaling by Erbb2,” “Glucose metabolism,” “Mitophagy,” and “Glycolysis” were used for GSEA. Normalized enrichment scores were calculated using the Kolmogorov-Smirnov statistics and nominal *p*-values were calculated using 1000 permutations. Analysis was performed by non-blinded individuals.

### Metabolomics

Mammary tumors were grown to a size of 5 × 5 mm. Tumors were randomly assigned time points using a random number generator. Prior to excision, mice were anesthetized using isoflurane (1–1.5% v/v) in room air in an induction chamber. The animal was transferred to a heated, sterile surgical field to maintain core body temperature and sterility. At each time point, mammary tumors were harvested from mice, flash-frozen, and pulverized into powder. Following excision, mice were immediately euthanized by isoflurane inhalation overdose. Euthanasia was confirmed using a bilateral thoracotomy.

Tumor samples and datasets were prepared and created by a blinded individual. Samples were analyzed for amino acids, acylcarnitines, and organic acids using a stable isotope dilution technique. For amino acids and acylcarnitine measurements, samples were prepared as previously described^79,80^ and levels were quantified using flow injection tandem mass spectroscopy. The results were captured using a Waters TQD mass spectrometer equipped with an Acquity^TM^ UPLC system and controlled by the MassLynx 4.1 operating system (Waters, Milford, MA). Further, for organic acid measurements, samples were quantified using methods previously described^81^ using Trace Ultra GC coupled to ISQ MS operating under Xcalibur 2.2 (ThermoFisher Scientific, Austin, TX). Metabolomic assays were completed by the Metabolomics Core Research Faculty within the Duke Molecular Physiology Institute.

Prior to analysis, the metabolite dataset was checked for missing values were imputed via a K-nearest neighbor algorithm, was normalized via log Pareto scaling, and underwent outlier detection. This resulted in the removal of one primary tumor treated with Etomoxir and 1 regression tumor treated with Etomoxir, all other groups contained five tumors. Comparisons of metabolites between time points are displayed as volcano plots where metabolites with both |Log_2_FoldChange| > 1 and *p*-values (*t*-test) <0.05 are considered significant. Analysis was performed by non-blinded individuals.

## Supplementary information

Supplementary Information

## Data Availability

RNA sequencing data comparing primary, regressing, and dormant fast-recurring MTB;TAN-derived tumor cell lines treated with and without Etomoxir is available online using the National Center for Biotechnology Information (NCBI) Short Read Archive (SRA) under BioProject ID PRJNA859129. All other datasets generated during the current study are available from the corresponding author on reasonable request.

## Code availability

All code generated during the current study are available from the corresponding author on reasonable request.
