# Supplementary material for: In vivo metabolic imaging identifies lipid vulnerability in a preclinical model of Her2+/Neu breast cancer residual disease and recurrence
Source: NPJ Breast Cancer. 2022 Sep 26;8:111. doi: 10.1038/s41523-022-00481-3 (PMC9512922; doi:10.1038/s41523-022-00481-3)
Supplement: Supplementary file 1 — Supplementary Information [file 41523_2022_481_MOESM1_ESM.pdf]

# In vivo metabolic imaging identifies lipid vulnerability in a preclinical model of Her2+/Neu breast cancer residual disease and recurrence

## Authors

Megan C. Madonna[1,*], Joy E. Duer[2], Brock J. McKinney[3], Enakshi D. Sunassee[1], Brian T. Crouch[1], Olga Ilkayeva[4], Matthew D. Hirschey[3,4,5], James V. Alvarez[3], Nirmala Ramanujam[1,3]

## Affiliations

[1] Department of Biomedical Engineering, Duke University, Durham, NC, USA

[2] Duke University Trinity College of Arts and Sciences, Durham, NC, USA

[3]Department of Pharmacology & Cancer Biology, School of Medicine, Duke University, Durham, NC USA

[4]Duke Molecular Physiology Institute and Sarah W. Stedman Nutrition and Metabolism Center, Durham, NC, USA

[5]Department of Medicine, Division of Endocrinology, Metabolism, and Nutrition, Duke University Medical Center, Durham, NC, USA

## Corresponding Author

*Department of Biomedical Engineering

1427 FCIEMAS, 101 Science Dr.

Campus Box 90281

Durham, NC 27708-0281

Phone: 919-660-8473

Fax: 919-684-4488
Email: megan.madonna@duke.edu

**Supplementary Figure 1**

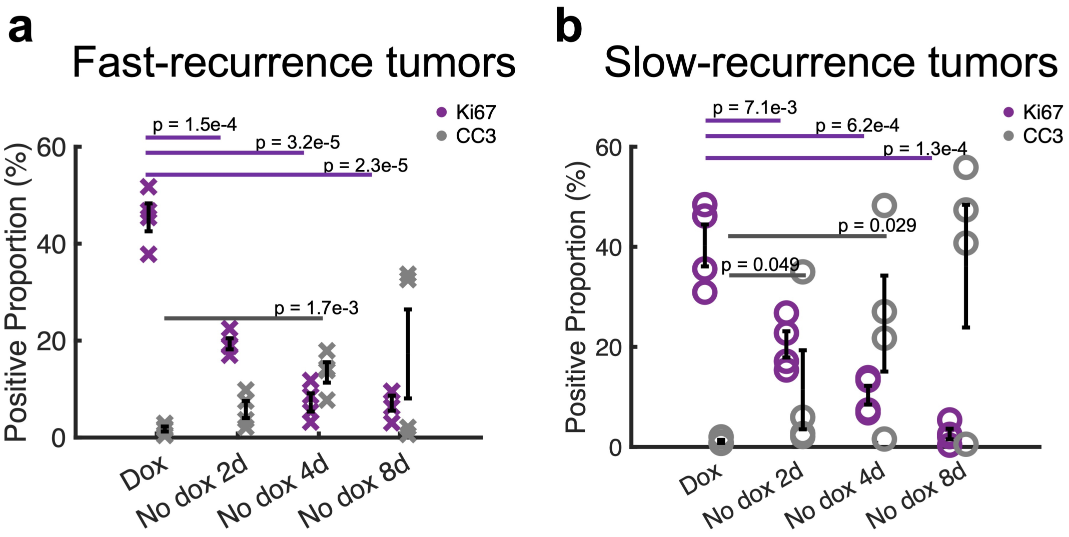


**Supplementary Figure 1: Quantification of immunohistochemistry results confirm changes in proliferation and apoptosis.** Ki67 and cleaved caspase 3 (CC3) were imaged from sectioned primary (+dox) and regression (No dox 2, 4, 8 days) for (**a**) fast-recurrence tumors and (**b**) slow-recurrence tumors. Ki67 and CC3 proportions were quantified by binary thresholding each cell as either positive or negative for staining. Additionally, statistical analysis using a one-way ANOVA followed by a Tukey test for multiple comparisons shows the highest proportion of Ki67 positive cells in primary tumors (Dox) and an increase in CC3 positive cells in regressing cells (No dox). n = 4 tumors. Error bar = standard error of the mean**.**

# Supplementary Figure 2

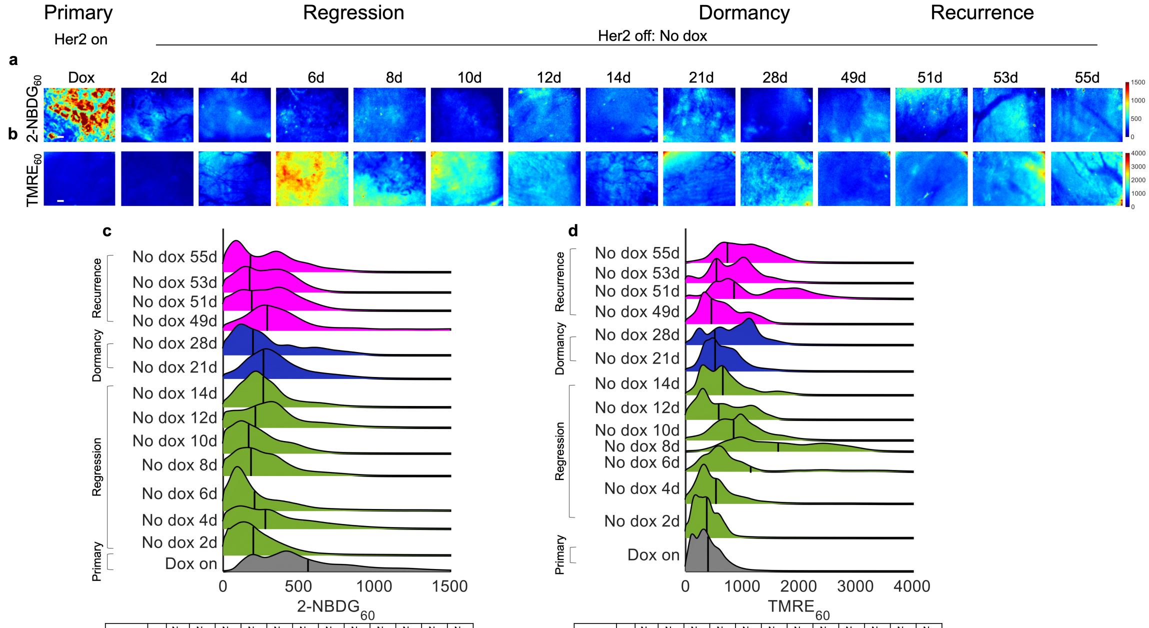


| | Dox | No Dox 2d | No Dox 4d | No Dox 6d | No Dox 8d | No Dox 10d | No Dox 12d | No Dox 14d | No Dox 21d | No Dox 28d | No Dox 49d | No Dox 51d | No Dox 53d |
|---|---|---|---|---|---|---|---|---|---|---|---|---|---|
| No Dox 2d | 0.024 | | | | | | | | | | | | |
| No Dox 4d | NA | NA | | | | | | | | | | | |
| No Dox 6d | 0.035 | NA | NA | | | | | | | | | | |
| No Dox 8d | 0.020 | NA | NA | NA | | | | | | | | | |
| No Dox 10d | 0.032 | NA | NA | NA | NA | | | | | | | | |
| No Dox 12d | NA | NA | NA | NA | NA | NA | | | | | | | |
| No Dox 14d | 0.048 | NA | NA | NA | NA | NA | NA | | | | | | |
| No Dox 21d | NA | 0.021 | NA | 0.030 | NA | 0.042 | NA | NA | | | | | |
| No Dox 28d | NA | NA | NA | NA | NA | NA | NA | NA | NA | | | | |
| No Dox 49d | NA | 0.010 | NA | 0.037 | NA | 0.032 | NA | NA | NA | NA | | | |
| No Dox 51d | NA | NA | NA | NA | NA | NA | NA | NA | NA | NA | NA | | |
| No Dox 53d | NA | NA | NA | NA | NA | NA | NA | NA | NA | NA | NA | NA | |
| No Dox 55d | NA | NA | NA | NA | NA | NA | NA | NA | NA | NA | NA | NA | NA |

| | Dox | No Dox 2d | No Dox 4d | No Dox 6d | No Dox 8d | No Dox 10d | No Dox 12d | No Dox 14d | No Dox 21d | No Dox 28d | No Dox 49d | No Dox 51d | No Dox 53d |
|---|---|---|---|---|---|---|---|---|---|---|---|---|---|
| No Dox 2d | NA | | | | | | | | | | | | |
| No Dox 4d | NA | NA | | | | | | | | | | | |
| No Dox 6d | 0.024 | 0.010 | NA | | | | | | | | | | |
| No Dox 8d | 0.001 | 0.001 | 0.001 | 0.037 | | | | | | | | | |
| No Dox 10d | 0.001 | 0.001 | 0.0060 | NA | 0.0230 | | | | | | | | |
| No Dox 12d | NA | NA | NA | NA | 0.001 | NA | | | | | | | |
| No Dox 14d | NA | 0.036 | NA | NA | 0.003 | 0.042 | NA | | | | | | |
| No Dox 21d | NA | 0.049 | NA | NA | 0.002 | NA | NA | NA | | | | | |
| No Dox 28d | 0.009 | 0.008 | NA | NA | 0.025 | NA | NA | NA | NA | | | | |
| No Dox 49d | NA | NA | NA | NA | 0.013 | NA | NA | NA | NA | NA | | | |
| No Dox 51d | 0.001 | 0.001 | 0.031 | NA | NA | NA | NA | NA | NA | NA | NA | | |
| No Dox 53d | 0.004 | 0.002 | NA | NA | 0.031 | NA | NA | NA | NA | NA | NA | NA | |
| No Dox 55d | 0.001 | 0.001 | 0.008 | NA | NA | NA | NA | 0.038 | NA | NA | NA | NA | NA |

**Supplementary Figure 2: Metabolic imaging following Her2 downregulation in tumors with a short period of dormancy.** Representative images of (**a**) 2-NBDG$_{60}$ and (**b**) TMRE$_{60}$ in a mammary tumor for Her2 on (+ dox) (n = 10) and Her2-off imaged every other day for 14 days (n = 10), during sustained growth arrest (No dox 21 and 28 days dox) (n = 8), and during regrowth (No dox 49-55 days) (n = 7). Scale bar = 200 µm. (**c**) 2-NBDG$_{60}$ and (**d**) TMRE$_{60}$ PDF ridgeline plots for Her2 on (+ dox) or Her2 off (- dox) across all pixels and all mice at each time point. Vertical lines superimposed on PDF curves report the average fluorescence. Statistical comparisons were performed using a Kolmogorov-Smirnov test; NS = not significant.

**Supplementary Figure 3**

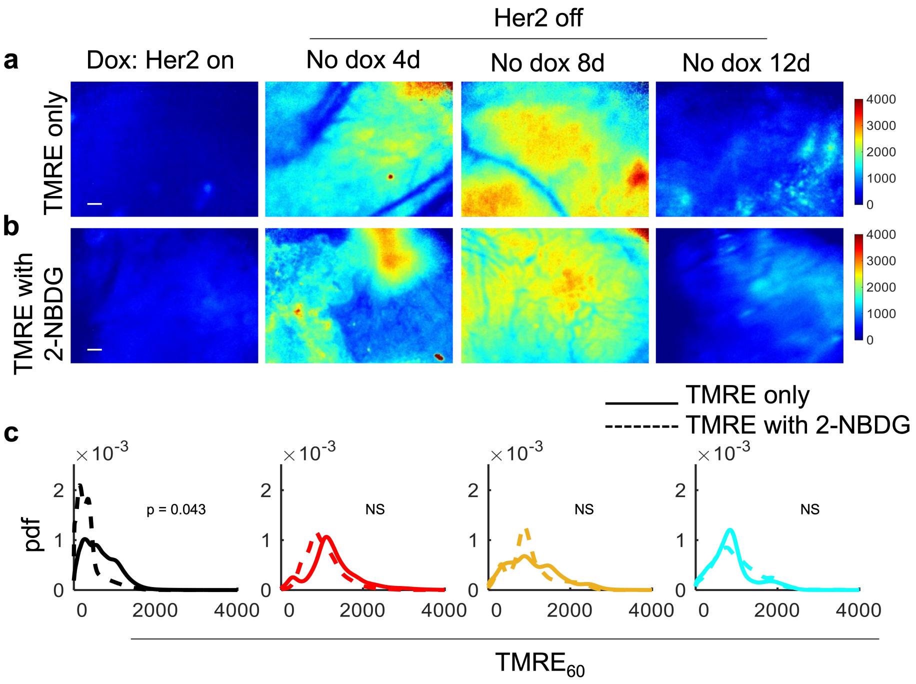


**Supplementary Figure 3: Simultaneous imaging does not mask metabolic changes following Her2 downregulation.** Primary (Her2 on) and Her2 downregulated tumors in mammary window chambers were imaged following TMRE only or TMRE and 2-NBDG injections. (**a**) Representative $TMRE_{60}$ images following TMRE only injection from mammary tumors for Her2 on (+ dox) (n = 10 mice) or Her2-off (4, 8, and 12 days after dox was removed, n = 12, 10, 7, respectively). (**b**) Representative $TMRE_{60}$ images following TMRE + 2-NBDG injection from mammary tumors for Her2 on (+ dox) or Her2-off (4, 8, and 12 days after dox was removed) (all groups; n = 10 mice). Scale bar = 200 µm. (**c**) $TMRE_{60}$ PDFs for Her2 on (+ dox) or Her2 off (- dox) across all pixels and all mice in each group. Statistical comparisons were performed using a Kolmogorov-Smirnov test; NS = not significant.

## Supplementary Figure 4

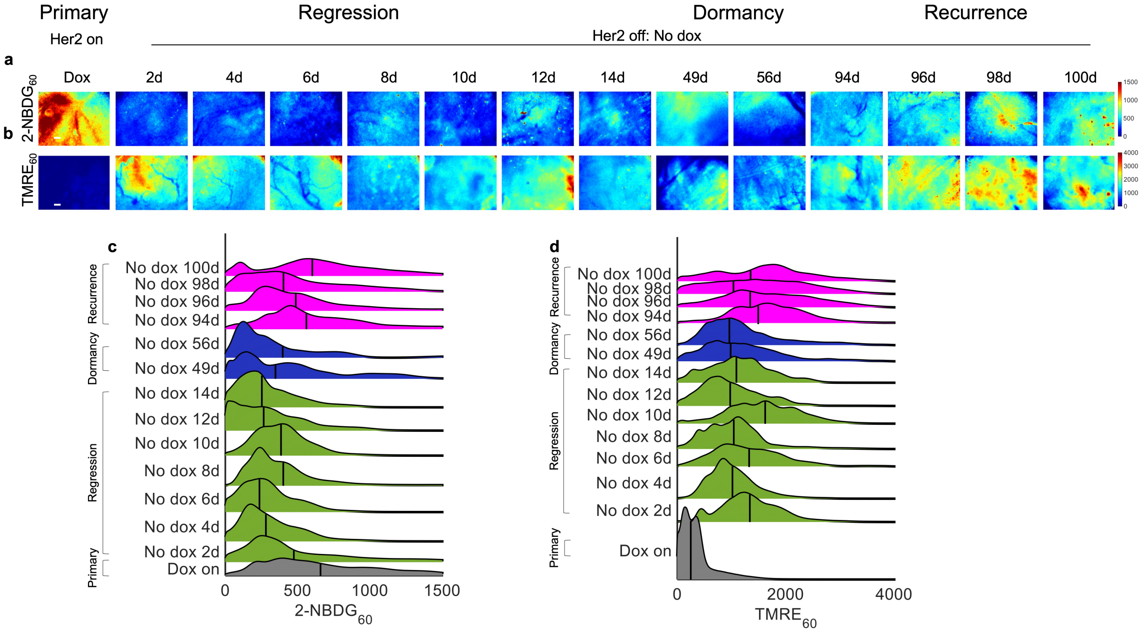


| | Dox | No Dox 2d | No Dox 4d | No Dox 6d | No Dox 8d | No Dox 10d | No Dox 12d | No Dox 14d | No Dox 49d | No Dox 56d | No Dox 94d | No Dox 96d | No Dox 98d |
|---|---|---|---|---|---|---|---|---|---|---|---|---|---|
| No Dox 2d | NA | | | | | | | | | | | | |
| No Dox 4d | 0.007 | NA | | | | | | | | | | | |
| No Dox 6d | 0.006 | NA | NA | | | | | | | | | | |
| No Dox 8d | 0.005 | NA | NA | NA | | | | | | | | | |
| No Dox 10d | 0.001 | NA | NA | NA | NA | | | | | | | | |
| No Dox 12d | 0.014 | NA | NA | NA | NA | NA | | | | | | | |
| No Dox 14d | 0.007 | NA | NA | NA | NA | NA | NA | | | | | | |
| No Dox 49d | NA | NA | NA | NA | NA | NA | NA | NA | | | | | |
| No Dox 56d | NA | NA | NA | NA | NA | NA | NA | NA | NA | | | | |
| No Dox 94d | NA | NA | 0.002 | 0.008 | 0.004 | NA | 0.012 | 0.004 | NA | NA | | | |
| No Dox 96d | NA | NA | NA | NA | NA | NA | NA | NA | NA | NA | NA | | |
| No Dox 98d | NA | NA | NA | NA | NA | NA | NA | NA | NA | NA | NA | NA | |
| No Dox 100d | NA | NA | 0.002 | 0.002 | 0.001 | 0.022 | 0.009 | 0.006 | NA | 0.033 | NA | NA | NA |

| | Dox | No Dox 2d | No Dox 4d | No Dox 6d | No Dox 8d | No Dox 10d | No Dox 12d | No Dox 14d | No Dox 49d | No Dox 56d | No Dox 94d | No Dox 96d | No Dox 98d |
|---|---|---|---|---|---|---|---|---|---|---|---|---|---|
| No Dox 2d | 0.001 | | | | | | | | | | | | |
| No Dox 4d | 0.001 | 0.035 | | | | | | | | | | | |
| No Dox 6d | 0.001 | NA | NA | | | | | | | | | | |
| No Dox 8d | 0.001 | 0.040 | NA | NA | | | | | | | | | |
| No Dox 10d | 0.001 | NA | 0.005 | NA | 0.009 | | | | | | | | |
| No Dox 12d | 0.001 | 0.032 | NA | NA | NA | 0.008 | | | | | | | |
| No Dox 14d | 0.001 | NA | NA | NA | NA | 0.049 | NA | | | | | | |
| No Dox 49d | 0.001 | NA | NA | NA | NA | NA | NA | NA | | | | | |
| No Dox 56d | 0.001 | NA | NA | NA | NA | NA | NA | NA | NA | | | | |
| No Dox 94d | 0.001 | 0.045 | 0.001 | NA | 0.005 | NA | 0.003 | 0.017 | NA | 0.018 | | | |
| No Dox 96d | 0.001 | NA | 0.006 | NA | 0.018 | NA | 0.015 | 0.046 | NA | NA | NA | | |
| No Dox 98d | 0.001 | NA | 0.022 | NA | 0.049 | NA | 0.031 | NA | NA | NA | NA | NA | |
| No Dox 100d | 0.001 | NA | 0.005 | NA | 0.025 | NA | 0.016 | NA | NA | NA | NA | NA | NA |

**Supplementary Figure 4: Metabolic imaging following Her2 downregulation in tumors with a long period of dormancy.** Representative images of (**a**) 2-NBDG$_{60}$ and (**b**) TMRE$_{60}$ in a mammary tumor for Her2 on (+ dox) (n = 10) and Her2-off imaged every other day for 14 days (n = 10), during sustained growth arrest (No dox 49 and 56 days – dox) (n = 8), and during regrowth (No dox 94-100 days – dox) (n = 9). Scale bar = 200 µm. (**c**) 2-NBDG$_{60}$ and (**d**) TMRE$_{60}$ PDF ridgeline plots for Her2 on (+ dox) or Her2 off (-dox) across all pixels and all mice at each time point. Vertical lines superimposed on PDF curves report the average fluorescence. Statistical comparisons were performed using a Kolmogorov-Smirnov test; NS = not significant.

**Supplementary Figure 5**

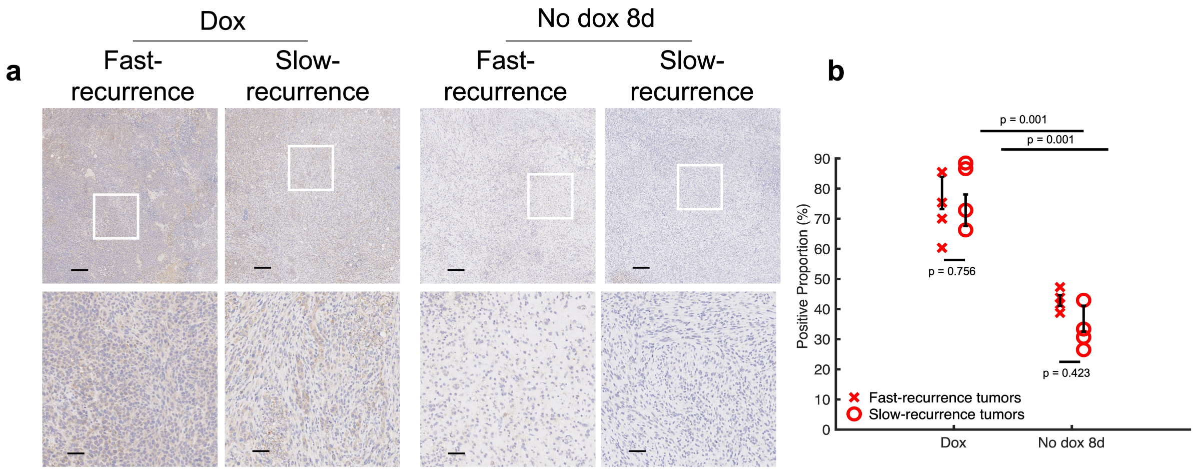


**Supplementary Figure 5: Immunohistochemistry and quantification of ATP5A1 as a surrogate for mitochondrial content comparison between fast and slow recurring tumors.** ATP5A1 (brown), a key subunit of the ATP synthase complex localized within the mitochondria, was (**a**) imaged and (**b**) quantified from sectioned primary (Dox) and regressing tumors. White boxes indicate magnified section shown in bottom panels. Scale bars represent 200μm (top panels) and 50μm (bottom panels). ATP5A1 proportions were quantified by binary thresholding each cell as either positive or negative for staining. Additionally, statistical analysis using a one-way ANOVA followed by a Tukey test for multiple comparisons shows no significant difference in ATP5A1 positive cells in slow- compared to fast-recurrence primary (Dox) tumors and slow- compared to fast-recurrence regression. n = 4 tumors. Error bar = standard error of the mean.

**Supplementary Figure 6**

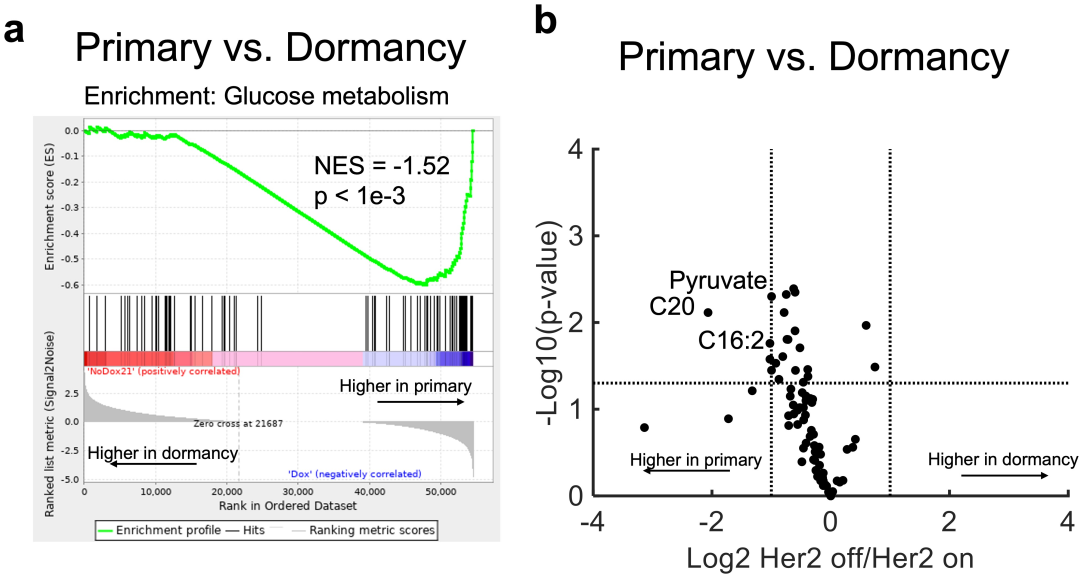


**Supplementary Figure 6: Changes in transcription and metabolite levels in dormant tumors compared to primary tumors**. (**a**) Gene set enrichment analysis (GSEA) of primary (dox) and dormant (no dox 21 days) tumors showing enrichment of the glucose metabolism gene set in primary tumors compared to dormant tumors, similar to results seen in primary tumors compared to regressing tumors. Enrichment scores were calculated using the KS statistic and p-values were calculated using permutation testing with 1000 permutations. (**b**) Volcano plot of fold-change in metabolite levels between dormant and primary tumors. Dashed lines represent $p<0.05$ via two-sided t-test and $|Log2FoldChange|>1$. Significantly different metabolites are labeled within graph. n = 5 per group.
